# An Assessment of China’s Digital Trade Development and Influencing Factors

**DOI:** 10.3389/fpsyg.2022.837885

**Published:** 2022-04-26

**Authors:** Yue Hu, Han Qian Zhou, Bin Yan, Zhou Zou, Yu’an Li

**Affiliations:** ^1^College of Science and Technology, Ningbo University, Cixi, China; ^2^School of International Trade and Economics, Ningbo University of Finance and Economics, Ningbo, China

**Keywords:** digital trade, Entropy-TOPSIS analysis method, principal component analysis method, information technology, digital transformation

## Abstract

The pattern and scale of commerce worldwide have been greatly transformed by the Fourth Industrial Revolution and technological advancement; digital trade has become the primary form of trade in the digital economy. On the basis of information network infrastructure, information technology level, digital industrialization level, and industrial digitalization level, this study establishes a comprehensive assessment system that applies an entropy–TOPSIS model to evaluate digital trade development level in China. The results indicate that digital trade in China was steadily growing between 2010 and 2019. A principal component analysis is conducted to identify factors affecting the digital trade development level in China. The analysis results suggest that Internet development, population income, industrial structure, payment convenience level, fixed asset investment, online transaction scale, and economic development all have positive effects on the digital trade development level in China, with payment convenience level having the greatest influence. By contrast, state intervention and degree of dependence on foreign trade have a negative effect on digital trade development.

## Introduction

The rapid advancement and widespread application of the Internet, big data, cloud computing, and artificial intelligence have enabled digital technologies to increasingly integrate with all sectors of the society and economy. The unprecedented development and expansion of the digital economy have made it a crucial force that redistributes strategic resources, restructures economy, and reshapes competition on a global scale. The advent of digital economy is the greatest change in human history since the Industrial Revolution; it has transformed the traditional relationship between individuals, corporations, and society (Quintanilla, 2015; Hopkins, 2021; Liebrecht et al., 2021). The digital economy is the manifestation of a nation’s comprehensive strength in the digital age and the engine that drives the development of a modern economic system. Major countries worldwide all emphasize the development of a digital economy; they have conceived diverse strategies and spared no effort to gain a competitive edge in a world that has been reshaped by the digital age. In 2020, the sum of value-added of the digital economies of 47 countries reached 32.6 trillion US dollars, which was equivalent to a 3% nominal growth from the previous year and accounted for 43.7% of their GDP. Among these countries, the United States was again at the top of the list, with the scale of its digital economy being 13.6 trillion US dollars. China occupied the second place, with the scale of its digital economy being 5.4 trillion US dollars. The third, fourth, and fifth places were occupied by Germany (2.54 trillion US dollars), Japan (2.48 trillion US dollars), and the United Kingdom (1.79 trillion US dollars), respectively. Notably, the digital economy plays a dominant role in the national economies of the United States, Germany, and the United Kingdom, accounting for more than 60% of their GDP. These statistics clearly show that the proportion of global digital economy in GDP is getting higher. Many countries have begun to attach the importance to development of digital economy and introduce relevant digital transformation strategies. Although the overall scale of China’s digital economy is larger than that of countries such as Germany and the United Kingdom, there is still much room for improvement.

The development of digital economy gave birth to digital trade, it creats new trade content and changes the traditional trade form. Driven by digital technologies, conventional trade in goods and services is upgrading and undergoing digitalization. Consequently, the continual growth of digital trade makes it the primary form of trade in a digital economy (Carlsson, 2004; Khumalo, 2010; Ma et al., 2018; Meltzer, 2019). The history of digital trade of China can be divided approximately into three stages, namely the electronic commerce stage (e-commerce; 1998–2012), which was characterized by informatization of commercial activities; the cross-border e-commerce stage (2013), which was a form of international commerce that relied on real-time gross settlement and involved the delivery of goods through international logistics services; and the digital trade stage (2014-present), which is manifested in the digitalization of the trade of physical goods as well as digital goods and services. In the digital trade history of China, e-commerce started at the beginning of the 21st century and entered a phase of rapid expansion in 2010, after which cross-border e-commerce became increasingly common, with the total value of e-commerce imports and exports reaching 26.993 billion US dollars in 2019 (a 38.3% growth from the previous year). Subsequently, the COVID-19 pandemic increased the growth of digital trade in all sectors. A comparison between the digital trade development in China and the rest of the world suggests that digital trade started relatively late in China. However, since the great expansion in 2010, the digital trade in China has maintained an annual growth of more than 20%, signaling a considerable potential for further development. Given that the data collected after 2020 are erratic because of the influence of the COVID-19 pandemic, the present study uses the data for the period from 2010 to 2019 to assess the development of digital trade in China and analyze the factors affecting (Jiao and Sun, 2021). Therefore, this study is the first to support the use of highly subjective assessment systems and successfully establish an objective assessment system based on the basis of indices supported by reliable data; the present study demonstrates an innovative application of the entropy–TOPSSI model for the assessment of a country’s digital trade development level. The combination of the entropy weight method and Technique for Order Preference by Similarity to Ideal Solution (TOPSIS) model leads to more objective assessments that serve as an example for the researchers in digital trade field.

## Literature Review

### Digital Trade

At the date of writing, the term *digital trade* still lacks a generally accepted definition; its definition varies considerably among organizations, institutions, and countries. Governments worldwide and their associated agencies initially regarded digital trade as a synonym of e-commerce. The World Trade Organization (WTO) defined e-commerce as the “production, distribution, marketing, sale or delivery of goods and services by electronic means” in 1988, and for years, this was accepted as the definition of digital trade. This changed in 2013 when the United States International Trade Commission (USITC) presented an official definition for digital trade in *Digital Trade in the United States and Global Economies, Part 1*, which was supplemented and refined in two follow-up reports published in 2014 and 2017. In 2017, the Organization for Economic Co-operation and Development (OECD) maintained that, although the definition of digital trade is still disputed, a consensus was forming. Subsequently, the OECD jointly published the *Handbook on Measuring Digital Trade* (1st edition) with the WTO and International Monetary Fund in 2020; this publication provides an official definition of the concept of digital trade. Table 1 lists the definitions of digital trade established by various organizations. On the basis of the conceptualization of digital trade, this study defines digital trade as a new trade model that delivers goods and services through methods that rely on continually advancing digital technologies (Lund and Manyika, 2016; Ma et al., 2019).

**TABLE 1:** Digital trade as defined by various organizations.

Organization (year)	Definition
The United States International Trade Commission (USITC) (2013)	The delivery of products and services through either fixed-line or wireless digital networks. This definition includes United States domestic commercial activity as well as international trade.
The United States International Trade Commission (USITC) (2014)	United States domestic commerce and international trade in which the Internet and Internet-based technologies play a particularly significant role in ordering, producing, or delivering products and services
The United States International Trade Commission (USITC) (2017)	The delivery of products and services over the Internet by firms in any industry sector.
OECD (2017)	Digitally enabled transactions of trade in goods and services that can either be digitally or physically delivered, and that involve consumers, firms, and governments.
OECD et al. (2020)	All trade that is digitally ordered and/or digitally delivered.

Digital trade has been a significant driving force behind the economic growth of countries worldwide. Product digitalization and digital transactions have expanded the scope of commodity transactions, which changes not only the form of trade but also the pattern of trade flows (Lund and Manyika, 2016; González and Jouanjean, 2017). Digital technologies can reduce the market cost for import and export trade and enhance trade efficiency (Meltzer, 2019). However, because digital trade has yet to mature, the lack of generally accepted rules between countries results in barriers to digital trade. Numerous researchers have investigated such barriers from the perspective of data privacy and protection requirements (Weber, 2010; Janow and Mavroidis, 2019; Wolfe, 2019), demands for localization (Cory, 2017), and intellectual property infringement (Meltzer, 2016). Other researchers have analyzed the rules for digital trade established through trade agreements such as those established by the WTO, the General Agreement on Tariffs and Trade, the Trade Facilitation Agreement, and the *Trans-*Pacific Partnership (Burri, 2012; Azmeh and Foster, 2016; Aaronson, 2018; Meltzer, 2019).

### Application of Entropy-Technique for Order Preference by Similarity to Ideal Solution

Entropy, a concept that originated from the field of thermodynamics, has been widely applied in the assessment of sustainable development and socioeconomics. The entropy weight method involves the calculation of the entropy value of each index based on the variance of an index, such that the obtained entropy values can be used as weights for the indices. In this method, the weigh selection process is highly scientific because it produces objective assessment results and prevents human factors from affecting index weights (Da Silva et al., 2010). The TOPSIS model is one of the most popular methods used for solving multicriteria discrete tasks, it is first proposed by Hwang and Yoon (1981), is a method that approximates an ideal solution through order preference, and it is based on the following concept: A virtual optimal solution and a virtual worst solution are established in a target space to determine the relative proximity of a solution to the ideal solution on the basis of how close this particular solution is to the positive ideal solution (i.e., the optimal solution) and how far it is from the negative ideal solution (i.e., the worst solution). The relative proximity of a solution to the ideal solution is expressed on a scale of 0 to 1, and solutions are ranked according to the value that is obtained by each solution; a greater value denotes a closer proximity to the ideal solution (Hwang and Yoon, 1981). Because of its simplicity, this method is widely applied in various fields, but it does not consider the status of solutions that appear on the perpendicular bisector of the line linking the positive ideal solution to the negative ideal solution (Opricovic and Tzeng, 2004; Jahanshahloo et al., 2006; Krohling and Pacheco, 2015). The present study combines the entropy weight method with the TOPSIS model to take advantage of the objective weighing practice of the entropy weight method and the ideal solution selection strategy of the TOPSIS model. As a matter of fact, Entropy-TOPSIS methods are used to solve complex decision-making issues in various areas. They are also adopted in issues related to the digitization of economies and societies (Table 2). However, so far the methods have not been used to assess the level of digital trade.

**TABLE 2:** Summary of applications of Entropy-TOPSIS methods to solve different problems.

Author (year)	Article	Methods
Brodny and Tutak, 2021a	Assessing the level of digitalization and robotization in the enterprises of the European Union Member States	TOPSIS
Brodny and Tutak, 2021b	Assessing the level of digital maturity of enterprises in the Central and Eastern European countries using the MCDM and Shannon’s entropy methods	TOPSISMOORA VIKOR
Yuan and Song (2021)	Evaluating technology innovation capabilities of companies based on entropy- TOPSIS: the case of solar cell companies	Entropy-TOPSIS
Cho and Dong-Jin (2021)	An Analysis of Competitiveness to Hold International Conferences by Regions in South Korea using Entropy-TOPSIS	Entropy-TOPSIS
Beyaz and Yıldırım (2020)	A Multi-criteria Decision-Making Model for Digital Transformation in Manufacturing	TOPSIS
Joonho (2020)	Analysis of Logistics Competitiveness of Pilot Free Trade Zones in China: Application of ENTROPY-TOPSIS	Entropy-TOPSIS
Watrobski et al. (2016)	Multistage performance modeling in digital marketing management	TOPSIS
Kim (2016)	A Study on Competitiveness Analysis of Ports in Korea and China by Entropy Weight TOPSIS	Entropy-TOPSIS

A literature review indicates that researchers are increasingly focusing on digitalization. However, the literature review also reveals that few researchers have applied the concept of weighting in their assessments (Schumacher et al., 2016; Bibby and Dehe, 2018; Pacchini et al., 2019). Assigning weights to indices is recommended because it negates subjectivity. As of the time of writing, no study has applied an entropy–TOPSIS model to assess digital trade development level. Therefore, the present study is the first to use the entropy–TOPSIS model to explore the digital trade development level in China.

## Research Model

### Index Selection and Source of Data

In *Digital Trade in the United States and Global Economies, Part 2* (2017), the USITC followed a line of thought that addresses digital trade from two statistical perspectives, namely (1) the analysis of the statistics on goods and services in digital trade and (2) the analysis of the statistics on the broadband data flow on the Internet. Ma et al. (2018) argues that digital trade is a new form of commercial activity that employs an information network infrastructure as a platform and realizes the delivery of goods or services through information and communication technology. Lan and Dou (2019) regards the degree of openness of a digital trade industry as a key dimension in the assessment of digital trade development level. Zhang and Zheng (2020) proposes trade potential as a key index for digital trade development level, and they proposed the use of GDP per capita and market openness as indices for assessing trade potential. Yang (2020) suggests that scientific research expenditure and business environment can be used to assess digital trade development level. Liu et al. (2020) suggests that the level of regional economic growth, degree of dependence on foreign capital, degree of state intervention, level of human capital, and resident income level should all be considered key indices in the field of digital economy.

On the basis of the literature review and the definitions of digital trade, the present study selects information network infrastructure, level of information technology, level of digital industrialization, and level of industrial digitalization as the four primary indices, which are further divided into 12 secondary indices (Table 3). The data analyzed are mainly extracted from the *China Statistical Yearbook* (2011–2020), *China Statistical Yearbook of the Tertiary Industry* (2011–2020), and *China’s E-commerce* (2011–2019). The data that are incomplete or missing are supplemented by data acquired through a trend analysis.

**TABLE 3:** Indices for digital trade development level.

Primary	Secondary
Information network infrastructure	Number of IPv4 addresses (unit: 10,000)
	Number of broadband Internet access ports (unit: 10,000)
	Number of mobile phone users (unit: 10,000)
Level of information technology	R&D in information and communication industry (unit: 10,000 RMB)
	Number of patent applications in information technology industry
	Number of employees in information technology industry (unit: 10,000)
Level of digital industrialization	Revenue of the software industry (unit: 100 million RMB)
	Total business volume of telecommunications industry (unit: 100 million RMB)
	Number of corporations that have completed corporate informatization
Level of industrial digitalization	Trade volume in online retail market (unit: trillion RMB)
	E-commerce service industry (unit: 100 million RMB)
	Trade volume in cross-border e-commerce industry (unit: trillion RMB)

### Assessment Method and Calculation Process

This study applies the entropy–TOPSIS model to compute and analyze the digital trade development level in China. Specifically, the entropy weight method is used to determine the weight of each index, after which a comprehensive assessment of the indices is performed using the TOPSIS model. The entropy–TOPSIS model combines the objective weighing practice of the entropy weight method and the ideal solution selection strategy of the TOPSIS model to effectively eliminate the problems and errors related to human subjectivity. Compared with the use of the entropy weight method or the TOPSIS model alone, the proposed model is more rational and objective.

#### Determination of Weights

The assessment is assumed to involve *m* years and *n* indices, and *x*_*ij*_ denotes the *j*th index in the *i*th year, thus the initial matrix can be expressed as *X* = (*x*_*ij*_) (Da Silva et al., 2010; Delgado-Bonal and Marshak, 2019). To avoid the problem of different units being used, the indices are normalized. Through the entropy weight method (Hu, 2002; Huang and Zhang, 2017; Du, 2020), the entropy value *e*, utility value *d*, and entropy weight *w* of each index are obtained using the following equations:


$$X=\left(X_{i\,j}\right)=\left(\begin{array}{ccc} x_{11} & \cdots & x_{1\,n}\\ \vdots & \ddots & \vdots \\ x_{m\,1} & \cdots & x_{m\,n} \end{array}\right)$$

$$X_{i\,j}^{\prime }=\frac{\left(x_{i\,j}-m\,i\,n\,x_{i\,j}\right)}{m\,a\,x\,x_{i\,j}-m\,i\,n\,x_{i\,j}}$$

#### Computing Process

Step 1 Establish a weighted decision matrix.

Matrix *X’* and weight *w* are normalized to establish a weighted decision matrix, which is expressed using the following equation:


Y=(yi⁢j)n×m=(wjx)′i⁢jn×m

Step 2 Determine the distance between positive and negative ideal solutions


$$y_{j}^{+}=\max\left(y_{i\,j}\right),y_{j}^{\text{—}}=\min\left(y_{i\,j}\right)\left(j=1,2,3,\ldots ,m\right)$$

Step 3 Calculate the distance of individual solutions to positive and negative ideal solutions


$$\begin{array}{c} S_{i}^{+}=\sum_{j=1}^{\text{N}}\left\{y_{j}^{+}\,\ln\frac{y_{j}^{+}}{y_{i\,j}}+\left(1-y_{j}^{+}\right)\,\ln\frac{1-y_{j}^{+}}{1-y_{i\,j}}\right\}\\ S_{i}^{-}=\sum_{j=1}^{\text{N}}\left\{y_{j}^{-}\,\ln\frac{y_{j}^{-}}{y_{i\,j}}+\left(1-y_{j}^{-}\right)\,\ln\frac{1-y_{j}^{-}}{1-y_{i\,j}}\right\} \end{array}$$

Step 4 Calculate the relative proximity of individual solutions


$$C_{i}=S_{i}^{-}/\left(S_{i}^{+}+S_{i}^{-}\right)$$

For the comprehensive index *C*_*i*_, a high value indicates that the assessed target (the *i*th solution) is closer to the positive ideal solution and hence a more favorable solution. The *C*_*i*_ value of each target (2010–2019) is calculated similarly by applying the entropy–TOPSIS model.

## Results

**FIGURE 1:**
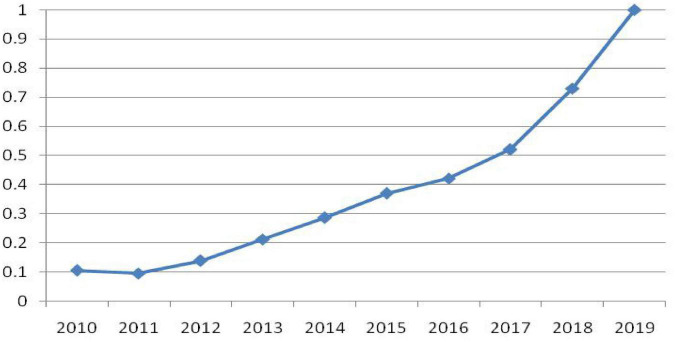
Digital trade development level in China from 2010 to 2019.

The present study can be divided into two stages. The first is the assessment of digital trade development level in China (Figure 1) through the establishment of indices (Table 3). The second is the analysis of the factors affecting China’s digital trade development level based on the findings of the first stage.

### Assessment of Digital Trade Development Level in China

By applying the entropy–TOPSIS model, weight method is first used to determine the weight of each index (Table 4). Subsequently, the TOPSIS model is employed for comprehensive analysis.

**TABLE 4:** Entropy weights obtained through entropy weight method.

Index	Entropy value *e*	Utility value *d*	Entropy weight *w*
Number of IPv4 addresses (unit: 10,000)	0.9561	0.0439	2.80%
Number of broadband Internet access ports (unit: 10,000)	0.8688	0.1312	8.37%
Number of mobile phone users (unit: 10,000)	0.9194	0.0806	5.14%
R&D in information and communication industry (unit: 10,000 RMB)	0.8986	0.1014	6.47%
Number of patent applications in information technology industry	0.895	0.105	6.69%
Number of employees in the information technology industry (unit: 10,000)	0.8965	0.1035	6.60%
Revenue of software industry (unit: 100 million RMB)	0.8894	0.1106	7.05%
Total business volume of telecommunications industry (unit: 100 million RMB)	0.7098	0.2902	18.50%
Number of corporations completing corporate informatization	0.9012	0.0988	6.30%
Trade volume in online retail market (unit: trillion RMB)	0.83	0.17	10.84%
E-commerce service industry (unit: 100 million RMB)	0.8084	0.1916	12.21%
Trade volume in cross-border e-commerce industry (unit: trillion RMB)	0.8581	0.1419	9.05%

After determining the weight of each index, the digital trade development level in China is assessed (Table 5). The results reveal an increasing trend for digital trade in China between 2010 and 2019, with the comprehensive index for digital trade growing from 0.299 (2010) to 1 (2019), indicating a qualitative growth. Specifically, the trend increased steadily between 2010 and 2016 and exponentially between 2017 and 2019. Throughout this period, the continual intensification of digital transformation in China made its digital economy a key driving force behind China’s economic growth, and it contributed considerably to the development of digital trade in the country.

**TABLE 5:** Digital trade in China from 2010 to 2019.

Year	Distance to positive ideal solution *S+*	Distance to negative ideal solution *S−*	Relative proximity *C*
2010	0.299	0.036	0.106
2011	0.304	0.032	0.096
2012	0.29	0.047	0.139
2013	0.268	0.072	0.213
2014	0.243	0.097	0.286
2015	0.214	0.126	0.37
2016	0.21	0.153	0.421
2017	0.172	0.187	0.521
2018	0.09	0.244	0.73
2019	0	0.318	1

### Identification of Factors That Affect Assessment Indices

On the basis of the assessment findings, the present study further investigates the main factors that affect the digital trade development level in China. Wang (2016) references the digital trade governance experiences of developed countries and postulates that information infrastructure, technical reserve, consumer market, and policy environment can substantially influence the development of digital trade. The Digital Trade Restrictiveness Index, which was published in 2018 by the European Center for International Political Economy, covers four main cluster areas, namely fiscal restrictions, establishment restrictions, restrictions on data, and trading restrictions (Ferracane et al., 2018). In 2019, the OECD published the Digital Service Trade Restrictiveness Index, which established a framework for the monitoring of barriers that affect trade in digitally enabled services. The framework covers five areas, namely infrastructure and connectivity, electronic transactions, payment systems, intellectual property rights, and others (Ferencz, 2019; Vovchenko et al., 2022). Du (2020) asserts that the development of digital trade in China is crucially affected by China’s rapidly growing information industry and digital industry. Other researchers have maintained that factors such as culture, system, technology level, and business environment can affect the development of digital trade (Lu and Fu, 2018; Wang, 2020; Lin and Hyukku, 2021). Therefore, the present study adopts the following factors as the factors that affect the digital trade development level in China (Table 6): Internet development level, population income level, industrial structure, state intervention, international trade level, payment convenience level, fixed asset investment, scale of online transactions, and economic development level.

**TABLE 6:** Factors affecting digital trade development level in China.

Factor	Corresponding index
Internet development level	Internet penetration rate (%)
Population income level	Per capita disposable income (RMB)
Industrial structure	Proportion of tertiary industry in GDP (%)
State intervention	Proportion of technology spending in GDP (%)
International trade level	Degree of dependence on foreign trade (%)
Payment convenience level	Online payment through non-bank payment institutions (100 million RMB)
Fixed asset investment	Fixed asset investment by information transfer, software, and information technology industries (100 million RMB)
Scale of online transactions	Number of online consumers (100 million)
Level of economic development	GDP (trillion RMB)

### Principal Components Analysis

Information consolidation is conducted using SPSSAU for principal components analysis. The first step of the principal components analysis is to determine whether the data on hand are adequate for principal components analysis. This is achieved by performing a Kaiser–Meyer–Olkin (KMO) test and a Bartlett’s test. The KMO test examines the correlation between variables, and obtaining a value of < 0.5 through the test indicates that the data are inadequate for principal components analysis; by contrast, a value of between 0.5 and 0.6 indicates moderate adequacy for analysis, and a value of > 0.6 indicates adequacy for analysis. For the Bartlett’s test, a significance of < 0.01 indicates that the data are adequate for principal components analysis (Jolliffe and Cadima, 2016). Table 7 reveals a KMO value of 0.792 (>0.6), indicating that the data are adequate for principal components analysis. For the Bartlett’s test, a *p* value of 0 is obtained, indicating that the data are adequate for principal components analysis.

**TABLE 7:** Results of Kaiser–Meyer–Olkin (KMO) test and Bartlett’s test.

KMO test		0.792
**Bartlett’s test**	**Approx. chi-square**	**210.317**
	
	df	36
	P	0

Tables 8, 9 present the three extracted principal components, which account for 91.543, 7.438, and 0.677% of the variance and explain 99.658% of the cumulative variance. Because the three principal components explain 99.658% of the variance, they are selected for principal components analysis.

**TABLE 8:** Variance results.

Eigenvalue			Principal components		
Eigenvalue	% of variance	Cumulative %	Eigenvalue	% of variance	Cumulative %
8.239	91.543	91.543	8.239	91.543	91.543
0.669	7.438	98.981	0.669	7.438	98.981
0.061	0.677	99.658	0.061	0.677	99.658
0.019	0.207	99.865	–	–	–
0.008	0.092	99.957	–	–	–
0.003	0.03	99.987	–	–	–
0.001	0.01	99.997	–	–	–
0	0.003	99.999	–	–	–
0	0.001	100	–	–	–

**TABLE 9:** Component score coefficient matrix.

Index	Component		
	F1	F2	F3
Internet penetration rate (X1)	0.12	0.067	2.25
Per capita disposable income (X2)	0.12	0.178	0.471
Proportion of tertiary industry in GDP (X3)	0.12	−0.126	−1.356
Proportion of technology spending in GDP (X4)	0.089	−1.009	0.534
Degree of dependence on foreign trade (X5)	−0.119	0.277	1.453
Online payment through non-bank payment institutions (X6)	0.112	0.539	−0.889
Fixed asset investment by information transfer, software, and information technology industries (X7)	0.12	0.097	−1.748
Number of online consumers (X8)	0.121	0.113	0.634
GDP (X9)	0.12	0.19	1.625

According to Table 9, the three principal component expressions are:


F⁢1=0.120*X⁢1+0.120*X⁢2+0.120*X⁢3+0.089*X⁢4-0.119*X⁢5+0.112*X⁢6+0.120*X⁢7+0.121*X⁢8+0.120*X⁢9

F⁢2=0.067*X⁢1+0.178*X⁢2-0.126*X⁢3-1.009*X⁢4+0.277*X⁢5+0.539*X⁢6+0.097*X⁢7+0.113*X⁢8+0.190*X⁢9

F⁢3=2.250*X⁢1+0.471*X⁢2-1.356*X⁢3+0.534*X⁢4+1.453*X⁢5-0.889*X⁢6-1.748*X⁢7+0.634*X⁢8+1.625*X⁢9

### Regression Analysis

A stepwise regression analysis in which *F1*, *F2*, and *F3* are the independent variables and *C*_*i*_ is the dependent variable is performed, and the analysis results reveal that *F3* is collinear with both *F1* and *F2*. Therefore, only *F1* and *F2* are retained in the model. The regression analysis model reveal that *R*^2^ = 0.967, thus *F1* and *F2* explain 96.7% of the variance in *C*_*i*_. The model passes the *F*-test (*F* = 103.503, *p* = 0.000 < 0.05), suggesting that it is effective.

The model can be expressed as follows:


$$C_{i}=0.276^{*}\,\mathrm{F1}+0.087^{*}\,\mathrm{F2}+0.388$$

A multicollinearity test reveals that the variance inflation factor values are all < 5, thus multicollinearity is not observed. Moreover, because the obtained *D-W* value is close to 2, model autocorrelation is ruled out, indicating that the model is robust. Finally, the regression coefficients of *F1* and *F2* are 0.276 (*t* = 13.718, *p* = 0.000 [<0.01]) and 0.087 (*t* = 4.340, *p* = 0.003 [<0.01]), respectively, suggesting that *F1* and *F2* significantly and positively affect *C*_*i*_.

The effects of the aforementioned indices on *C*_*i*_ can be more clearly expressed by substituting principal component expressions into the following regression equation:


$$C_{i}=\,0.039\,X\,1+0.049\,X\,2+0.022\,X\,3-0.063\,X\,4-0.009\,X\,5+0.077\,X\,6+0.042\,X\,7+0.043\,X\,8+0.05\,X\,9+0.388$$

The regression results indicate that Internet development level, population income level, industrial structure, payment convenience level, fixed asset investment, scale of online transactions, and economic development level all promote the development of digital trade in China, with payment convenience level having the most pronounced effect. By contrast, state intervention and degree of dependence on foreign trade negatively affect the development of digital trade. However, China’s dependence on foreign trade has decreased in the past decade. In 2010 the degree of dependence was 48.9%, and by 2019, this figure had dropped to 31.8%. The reduced role of the international economic cycle in China can be attributed to the country’s policy of expanding domestic demand to develop a strong domestic market. Therefore, the decrease in the degree of dependence on foreign trade can be inferred to negatively affect the development of digital trade.

## Discussion and Conclusion

This study applies the entropy–TOPSIS model and 12 indices to assess the digital trade development level in China (Figure 1 and Table 3). It also applies a principal components analysis (Tables 7–9) and a regression analysis (Table 10) to assess the factors that affect the digital trade development level in China. The conclusions drawn are as follows. (1) The development of digital trade in China exhibited a general increasing trend between 2010 and 2019, with the comprehensive index increasing from 0.299 (2010) to 1 (2019), indicating a qualitative growth. Specifically, the trend increased steadily between 2010 and 2016 and exponentially between 2017 and 2019. Through the entropy weight method, a weight of 18.5% is obtained for the total business volume of the telecommunications industry, suggesting that the telecommunications industry is a prerequisite for the development of digital trade. Moreover, the number of broadband Internet access ports, e-commerce service industry, trade volume in the online retail market, and trade volume in the cross-border e-commerce industry all receive weights that are greater than 8%, suggesting that the digital trade development level in China is reliant on the establishment of network infrastructure, e-commerce services, and cross-border e-commerce. (2) Internet development level, population income level, industrial structure, payment convenience level, fixed asset investment, scale of online transactions, and economic development level all facilitate the development of digital trade in China, with payment convenience level having the most pronounced effect. By contrast, state intervention and degree of dependence on foreign trade are revealed to have a negative effect on the development of digital trade.

**TABLE 10:** Results of stepwise regression analysis (*n* = 10).

	Unstandardized coefficient		Standardized coefficient	*t*	*p*	VIF	*R* ^2^	Adjusted *R*^2^	F
	B	Standard error	Beta						
Constant *C*	0.388	0.019	–	20.354	0.000	–	0.967	0.958	*F*(2,7) = 103.503, *p* = 0.000
F1	0.276	0.02	0.938	13.718	0.000	1			
F2	0.087	0.02	0.297	4.34	0.003	1			
Dependent variable: C_i_									
*D-W*: 1.673									

### Theoretical Implications

The findings of this study have three theoretical implications. First, in the integration of digital trade and digital economy theories, the present study is the first to support the use of highly subjective assessment systems and successfully establish an objective assessment system based on the basis of indices supported by reliable data; thus, the present study enriches the literature on digital trade development level. Most studies have focus on the barriers to digital trade (Weber, 2015; Meltzer, 2016; Cory, 2017; Janow and Mavroidis, 2019), rules for digital trade (Burri, 2012; Azmeh and Foster, 2016; Aaronson, 2018; Meltzer, 2019), and maturity level of digital trade (Brodny and Tutak, 2021b). To the author’s knowledge, no study has explored the overall trend of a country’s digital trade development. Second, this study investigates the factors that affect a country’s digital trade development on the basis of its assessment of digital trade development. It references previous studies to identify indices for digital trade development. The results indicate that, among the identified factors, payment convenience level has the most pronounced positive effect on the digital trade development in China, whereas state intervention and degree of dependence on foreign trade have a negative effect on the digital trade development in China. This finding can serve as excellent references for countries that are seeking to establish policies that facilitate the development of digital trade. Third, the present study demonstrates an innovative application of the entropy–TOPSSI model for the assessment of a country’s digital trade development level. Other studies have mostly used the TOPSIS model to evaluate socioeconomical phenomena (Watrobski et al., 2016; Beyaz and Yıldırım, 2020; Brodny and Tutak, 2021a). The combination of the entropy weight method and TOPSIS model leads to more objective assessments that serve as an example for the researchers in digital trade field.

### Practical Implications

From the viewpoint of upgrading conventional trade through digital transformation, the present study reveals that the development of a country’s digital trade is dependent on the establishment of infrastructure and cross-border e-commerce. Therefore, a government should improve the digital infrastructure of its country and emphasis Internet infrastructure as well as the infrastructure and technology for cross-border commerce; this strategy leads to the establishment of a hardware environment that is conducive to digital trade. Furthermore, the business environment for digital trade must be optimized. Governments should steadily promote the Internet Plus strategy, encourage conventional corporations to undergo digital transformation, establish platforms for digitalization, and help corporations to improve their digital management abilities. From the perspective of a business environment, a country’s Internet development level and payment convenience level can both affect the development of its digital trade. Therefore, governments should increase investments in digital technologies such as the Internet, big data, and artificial intelligence to create a business environment conducive to digital trade.

### Limitations and Future Research

The present study makes several useful conclusions. In particular, it reveals that digital trade in China is significantly and positively affected by payment convenience level but negatively affected by state intervention and degree of dependence on foreign trade. Nevertheless, the present study has several limitations. First, because digital trade is a new domain, researchers have investigated it from the perspectives of their respective fields. Moreover, the statistics on digital trade have not been standardized, making the comparison of data from different countries difficult. For this reason, the present study investigates the development of digital trade in a single country. Second, during the establishment of the comprehensive index for assessing digital trade development level, the relevant indices included in the present study are not exhaustive. Although an effort was made to adopt them to reflect all the most relevant areas related to digital trade, it is obvious that they do not fully describe the process. An increasing number of studies are exploring digital trade, thus the assessment of digital trade development level is expected to become increasingly comprehensive, thereby providing a reference for future studies to develop more accurate indices. Third, in the present study, the selection of the factors that affect digital trade development level is based on the results of a literature review and practical experience. Therefore, the theoretical foundation of the present study is weak. With the gradual standardization of statistical data, future studies on digital trade should be able to reliably compare the experiences of multiple countries in digital trade development and identify the causes for their differences.

## Data Availability Statement

The original contributions presented in the study are included in the article/supplementary material, further inquiries can be directed to the corresponding authors.

## Author Contributions

All authors contributed equally to the conception of the idea, implementing and analyzing the experimental results, wrote the manuscript, read, and approved the final manuscript. All authors contributed to the article and approved the submitted version.

## Conflict of Interest

The authors declare that the research was conducted in the absence of any commercial or financial relationships that could be construed as a potential conflict of interest.

## Publisher’s Note

All claims expressed in this article are solely those of the authors and do not necessarily represent those of their affiliated organizations, or those of the publisher, the editors and the reviewers. Any product that may be evaluated in this article, or claim that may be made by its manufacturer, is not guaranteed or endorsed by the publisher.
